# Construction Notes

**Published:** 1893-10-14

**Authors:** 


### CONSTRUCTION NOTES.

**New Eye Infirmary for Greenock.**

The memorial-stone of this institution was recently laid, the building being the generous gift of Mr. Anderson Rodger. It is designed in the cottage hospital style from the designs of Mr. J. B. Stewart, I.A., architect, of Greenock, and consists of two separate departments—the indoor (which has accommodation for twenty beds), for the treatment of all serious cases of disease or injury; and outdoor department, for the treatment of cases which do not require special nursing or operative interference. The hospital consists of fourteen apartments, and includes recreation-rooms for male and female patients, matron and servants' rooms, kitchen and offices, with washing-house and laundry, an operating-room, dispensary, and doctor's room. The institution will probably be opened in the spring.

**Cottage Hospital at Dartmouth.**

Designs for this new cottage hospital were accepted from Messrs. Tait and Harvey, architects, of Exeter. The building has been skilfully designed, and the general arrangements are most complete. The main wards will be 30 feet by 22 feet and 11 feet high. The wards face south and east, and are provided with a bath-room, scullery, and other accommodation. The main staircase will be constructed so that a stretcher can be carried up, and it will be in direct communication with the operating-room on the first floor. A side entrance will lead to the kitchen and offices, which will comprise all necessary accommodation. The operating-room opening on to the main staircase will be lighted from the top, and immediately opposite will be the separation ward. Three rooms will be provided for matron and servants. Wood block paving will be used on the ground floor, and the wards will be heated by means of open fireplaces, fitted with warm air ventilating grates.

